# Cognitive Control and Discourse Comprehension in Schizophrenia

**DOI:** 10.1155/2012/484502

**Published:** 2012-04-08

**Authors:** Megan A. Boudewyn, Cameron S. Carter, Tamara Y. Swaab

**Affiliations:** ^1^Department of Psychology and Center for Mind and Brain, University of California, Davis, One Shields Avenue, Davis, CA 95616, USA; ^2^Department of Psychiatry and Behavioral Sciences, Imaging Research Center, and Center for Neuroscience, University of California, Davis, Davis, CA 95616, USA

## Abstract

Cognitive deficits across a wide range of domains have been consistently observed in schizophrenia and are linked to poor functional outcome (Green, 1996; Carter, 2006). Language abnormalities are among the most salient and include disorganized speech as well as deficits in comprehension. In this review, we aim to evaluate impairments of language processing in schizophrenia in relation to a domain-general control deficit. We first provide an overview of language comprehension in the healthy human brain, stressing the role of cognitive control processes, especially during discourse comprehension. We then discuss cognitive control deficits in schizophrenia, before turning to evidence suggesting that schizophrenia patients are particularly impaired at processing meaningful discourse as a result of deficits in control functions. We conclude that domain-general control mechanisms are impaired in schizophrenia and that during language comprehension this is most likely to result in difficulties during the processing of discourse-level context, which involves integrating and maintaining multiple levels of meaning. Finally, we predict that language comprehension in schizophrenia patients will be most impaired during discourse processing. We further suggest that discourse comprehension problems in schizophrenia might be mitigated when conflicting information is absent and strong relations amongst individual words are present in the discourse context.

*“There is no “centre of Speech” in the brain any more than there is a faculty of Speech in the mind.*

*The entire brain, more or less, is at work in a man who uses language”*

William James

From The Principles of Psychology, 1890

*“The mind in dementia praecox is like an orchestra without a conductor”*

Kraepelin, 1919

## 1. Introduction

Impaired cognition across a wide range of cognitive domains is a pervasive feature of schizophrenia and is connected to poor functional outcome for patients [1, 2]. Of the cognitive deficits that have been observed in schizophrenia patients, language abnormalities are among the most salient and include disorganized speech as well as deficits in comprehension. However, there is no general consensus as to whether the cognitive impairments seen in schizophrenia can be attributed to a single disrupted mechanism, to multiple disrupted systems, or to low-level perceptual deficits.

Accounts of language deficits in schizophrenia might be divided into theories that focus on irregularities in semantic memory structure and functioning, and those that emphasize deficits in the ability to effectively use context [3]. Accounts that posit abnormalities in semantic memory in schizophrenia, such as the exaggerated spread of activation in a semantic network [4], are largely based on studies involving the processing of words in isolation. In contrast, accounts of high-level language processing have focused on impairments in the ability to build and maintain context as the root of language deficits in schizophrenia. Such deficits have been attributed to problems with general cognitive processes of enhancement and suppression in a prominent theory of discourse processing, the structure-building framework [5]. Kuperberg [6] has proposed an imbalance between semantic-memory-based processing systems and combinatorial mechanisms in schizophrenia, such that patients often rely to a greater extent on semantic-memory-based processing at the expense of the structured build-up of context. The interaction between the semantic-memory-processing stream and the combinatorial stream has been proposed to be influenced by cognitive control mechanisms [7]. According to this view, control mechanisms should therefore be recruited when integration demands are high.

In the remainder of this theoretically-oriented review paper we seek to increase understanding and highlight novel hypotheses related to impairments in aspects of language processing in schizophrenia. We will begin with a discussion of language comprehension in the healthy human brain and will highlight the potentially important role of cognitive control processes, especially during discourse comprehension. We will then review the literature on cognitive control deficits in schizophrenia. Finally, we will review evidence suggesting that schizophrenia patients are particularly impaired at processing meaningful discourse as a result of impaired control functions. We conclude that schizophrenia patients are impaired in discourse comprehension when control demands are particularly high, for example, when different aspects of language input conflict with one another.

## 2. Cognitive Control and High-Level Language Processing in Healthy Participants

Outside of the laboratory, natural language processing almost always involves a rich signal, featuring multiple connected sentences that each generates a meaningful context and also must be linked to the overall meaning provided by discourse context. For example, a sentence such as “Yesterday he went to the bank” may be interpreted as describing a person visiting a financial institution, yet that interpretation would be incorrect if the preceding discourse was about a person going to the side of a river. In short, discourse context is a complex web of information including word-level, sentence-level, and message-level meaning, as well as syntactic structure, previously stored background information, and other context information (e.g., speech-accompanying gestures used by the speaker). 

In order to construct a coherent meaning representation, language users must integrate these various sources of information, and some prominent models of language comprehension predict that this involves specific control mechanisms. As briefly discussed earlier in this review, one theory that proposes an important role of control mechanisms during discourse comprehension is the structure-building framework, which emphasizes suppression ability as critical to language comprehension [8–10]. According to this framework, incoming language input is mapped onto mental structures via the enhancement of relevant information and suppression of irrelevant information. When input fits well with the current structure, it will be incorporated into that structure. This process is referred to as mapping, which involves adding on to the developing representation of the text. Integration of new information into an existing representation places fewer demands on cognitive resources than the initial construction of a discourse representation; therefore, incoming input that is coherent with the previous context leads to faster processing. When input is less coherent with the existing structure, the comprehender will construct a new substructure in order to accommodate the new information into the overall representation (shifting). The creation of a new substructure has been labeled “shifting,” and it requires additional processing, which can slow down comprehension. A failure to effectively suppress irrelevant information that is activated by the incoming input (e.g., the financial-institution meaning of the word “bank” in the example above, when context-irrelevant) may result in the construction of excessive substructures [8–10]. The cognitive processes of mapping and shifting are thought to rely on control networks; inefficient mapping, and particularly shifting, has been argued to result in poor discourse comprehension in healthy adults, since this could lead to lingering activation of discourse-irrelevant information [8–10]. Excessive shifting could also result in the disorganized discourse representations that have been observed in schizophrenia patients [5].

Another model of language processing that recognizes a role of cognitive control mechanisms during language processing is the memory, unification, and control (MUC) model [11]. Briefly, according to the MUC model, language processing requires activation and retrieval of semantic and syntactic memory representations stored in left temporal cortex. Unification refers to the construction of a meaning representation involving multiple words (or sentences), using information about the words that were activated and retrieved. Unification processes are proposed to be mediated by the left inferior frontal cortex (Broca's complex). Finally, control processes are assumed to be mediated by the dorsolateral prefrontal cortex (DLPFC) and anterior cingulate cortex (ACC) and are recruited when attentional control is necessary, for example, during turn-taking in conversation and during the selection of situation-appropriate language in bilinguals [11]. (Other theories also suggest a direct role of cognitive control during processing of other aspects of the language input (for reviews, see [12, 13].)

 In sum, prominent theories of high-level language processing concur that comprehension is supported by control mechanisms. In addition, in the case of incoherent discourse, one or more sources of information may conflict (e.g., activation of the financial-institution meaning of the word bank would conflict with discourse-level context in which the side-of-the-river meaning is promoted), placing even greater demands on these control mechanisms to support comprehension.

## 3. Cognitive Control and Schizophrenia

Cognitive control is somewhat of an umbrella term in that it refers to domain-general processes that govern processing in other systems, including the allocation of attentional resources, conflict detection and resolution, the maintenance of task-relevant information, and inhibition. According to the Guided Activation Model, control mechanisms mediating top-down processing are based in the prefrontal cortex [14]. Specifically, the PFC maintains and represents task-relevant context, including goals, which serves to guide processing in relevant neural regions associated with task execution [14]. Deficits in this ability to maintain context in order to guide processing would be expected to result in a wide range of impairments across cognitive domains. Several theories postulate that impaired cognitive control might account for the widespread deficits in cognition seen in schizophrenia [15–18]. As noted above, alternative explanations suggest that deficits in sensory or perceptual systems may account for the cognitive deficits seen in schizophrenia (e.g., [19]). In the current paper, we adopt the approach outlined by Lesh and colleagues [18]; namely, that a single underlying deficit in prefrontally mediated control functions is at the heart of the broad range of cognitive deficits that have been observed in schizophrenia patients.

A detailed discussion of cognitive dysfunction in schizophrenia, and the evidence that suggests deficits in control mechanisms are the basis for this dysfunction, is beyond the scope of this review. However, it is important to note that there is substantial evidence suggesting a domain-general control deficit in schizophrenia patients and specifically showing reduced DLPFC activity accompanied by impaired behavioral performance in patients during tasks that are demanding of cognitive control (see [18] for a review), though a recent meta-analysis of executive-function tasks also showed that reduced DLPFC activation is present in schizophrenia whether performance is impaired or matched [20]. One salient example of empirical evidence for a control deficit in schizophrenia comes from a study by MacDonald and colleagues [21]. They presented never-medicated, first-episode schizophrenia patients, as well as never-medicated nonschizophrenia psychosis patients and healthy controls, with the AX-CPT task in an fMRI session [21]. The AX-CPT is a task that is specifically designed to tax cognitive control functions: subjects are instructed to press a button every time they see an “X” that immediately follows an “A” and to otherwise withhold their response. This type of trial (AX trial) is highly frequent, accounting for 70% of total trials; this leads to the tendency to respond to an X and to anticipate making a response after an A. Therefore, false alarms often occur in response to AY trials (where “Y” stands for any letter except “X”) as the context of “A” leads to the expectation of an upcoming response. Finally, BX trials (where “B” stands for any letter except “A”) are least frequent and are most demanding on the controlled maintenance of context, because participants must use the context of having just encountered a “B” in order to correctly inhibit their response to the “X”. Compared to controls and nonschizophrenia psychosis patients, never-medicated schizophrenic patients made more errors on BX trials [21]. fMRI results for BX trials for which controls responded appropriately showed increased activity in the DLPFC and also the posterior parietal areas compared to the less demanding AX trials. For the patient group, this same contrast showed that DLPFC activity was significantly reduced compared to controls and that reduced DLPFC activity was linked to increased disorganization symptoms in the schizophrenic individuals [21]. These results are consistent with several other studies that have also found reduced DLPFC activity corresponding to poor performance on control tasks in schizophrenia patients (e.g., [22–24]) as well as the results of a recent meta-analysis [20]. Importantly, the reduction in DLPFC activity found for schizophrenia patients compared to controls is most pronounced under control-demanding task conditions; when control demands are relatively low, DLPFC activation for patients has been shown to approximate that of controls (e.g., [23]) or even to exceed that of controls (e.g., [25, 26]).

## 4. Cognitive Control, High-Level Language, and Schizophrenia

Language dysfunction is a hallmark of schizophrenia, leading to the production of disorganized speech as well as deficits in language comprehension (for reviews, see [6, 27, 28]). However, schizophrenia is far from a homogenous disorder; symptoms vary across individuals and symptoms within an individual can vary over time as well. When present, common language phenomena observed in schizophrenia patients include tangentiality (jumping from topic to topic without providing obvious links in response to a question), derailment (disjointed speech that slips from topic to topic), incoherence (incomprehensible speech), and poverty of speech (reduction in the quantity of speech) [29, 30]. Although many of the features of language dysfunction in schizophrenia are observed in production at the discourse level (e.g., tangentiality and derailment), the bulk of the research on real-time language comprehension in schizophrenia patients has focused on the word- and sentence-levels of processing.

A prominent area of research on language comprehension in schizophrenia has been the influence of word-level meaning associations on the processing of incoming words (i.e., semantic priming paradigms). Results from these studies suggest that the activation and retrieval of stored meaning representations of words is relatively intact, and some studies even show larger-than-normal effects of semantic priming (e.g., [7, 31–33]). Interestingly, under “automatic” priming conditions (e.g., when targets follow closely after primes), schizophrenia patients show normal or exaggerated semantic priming effects but show smaller or absent effects when controlled processing is required, such as evaluation of the relation between prime and target [3, 33]. This pattern of results has been interpreted as an indication of semantic memory dysfunction in schizophrenia, specifically involving a faster and more extensive propagation of activation in semantic memory [4, 28, 34]. 

 At the sentence level, there is also a great deal of work showing various deficits in schizophrenia compared to healthy adults (e.g., [35–37]). Much of this work can be summarized as suggesting a deficit in the build-up and maintenance of context: compared to healthy controls, schizophrenia patients have been shown to be unable to benefit from linguistic context in a word-monitoring task [35] and to have difficulty using sentence context to select the context-appropriate meaning of an ambiguous word [36]. Sitnikova and colleagues [36] presented schizophrenia patients and healthy controls with sentences that biased towards either the dominant meaning of an ambiguous word (e.g., *bridge* as an architectural structure) or the subordinate meaning (*bridge* as a card game). The second clause of the sentences also contained a word that was semantically associated to the dominant meaning of the ambiguous word that had appeared earlier in the sentence (e.g.,… because the *river* had rocks in it, following either “diving was forbidden from the *bridge …*” or “The guests played *bridge *…”). Sitnikova and colleagues [36] measured the electrical activity of the brain as healthy controls and patients read the sentences. Event-related potentials (ERPs) were extracted for critical words that were either consistent or not with the preceding sentence contexts. Of particular relevance to this study is an ERP effect that has been labeled the N400. The N400 is a negatively deflecting ERP waveform that is modulated by semantic fit, such as relatedness to previous words and congruence or predictability given prior context (for a review, see [38]). Controls showed a decreased N400 amplitude for target words like *river* when they were appropriate given the previous sentence context (diving was forbidden from the bridge …) compared to when they were inappropriate (the guests played bridge …). However, patients' N400 effects to context-appropriate and context-inappropriate target words were indistinguishable, suggesting that patients were unable to benefit from context to suppress the irrelevant meaning of the ambiguous word (e.g., *river*) [36]. In contrast, the patient and control groups did not differ in their response to unambiguous context-congruent compared to incongruent words (e.g.,… the river had *rocks/cracks* in it …), such that both groups showed a larger N400 amplitude in response to incongruent words (*cracks*) than to congruent words (*rocks*) [36]. This set of results is suggestive of a specific impairment when the controlled use of linguistic context is required, such as to suppress context-inappropriate meanings of words, rather than of an overall lack of sensitivity or attention to language context. 

Further evidence supporting deficits in the use of linguistic context in schizophrenia comes from a 2006 study from Kuperberg and colleagues. Patients and controls were presented with syntactically well-formed sentences that contained animacy violations (e.g., for breakfast the eggs would only *eat* toast and jam). Previously, a P600 effect was found in healthy adults when comparing these types of sentences to those containing no animacy violation (e.g., for breakfast the boys would only *eat* toast and jam) [27]. Semantic violations typically elicit N400 effects, whereas P600 effects have traditionally been linked to syntactic manipulations (see [38] for a review). The so-called “semantic P600” found in healthy adults has been interpreted as reflective of conflict between the syntax-dictated sentence meaning (e.g., that the eggs were eating) and the aggregate meaning based on relations among individual words (e.g., that *eggs* and *eat* are typically combined such that the eggs are being eaten) [27, 39]. Interestingly, schizophrenia patients show a reduced semantic P600 effect relative to controls [37]. This pattern of results suggests that when strong semantic relations amongst individual words are in conflict with sentence-level meaning as dictated by syntax, schizophrenia patients appear to be overly influenced by the word-level relations. 

In contrast, very few studies have looked at online discourse comprehension in schizophrenia. As discussed earlier, several theories of high-level language comprehension predict that demands on cognitive control are high during discourse processing, as discourse is a rich and multilevel signal containing a great deal of information to maintain and integrate, all with the potential to generate conflict. Given a model of cognitive dysfunction in schizophrenia based on control deficits [18], clear difficulties in language processing might be expected at the discourse level. 

Indeed, offline studies of memory have shown that patients do not benefit from discourse organization to the same extent as controls, manifesting as a lack of improved text recall with increased text coherence [40–43]. Further, a recent electrophysiological study presented schizophrenia patients and healthy adults with three-sentence passages in which the individual sentences were highly related to one another, intermediately related, or unrelated [44]. For example, a highly related passage might describe two characters as having an argument in the first sentence, as hitting each other in the second sentence, and as having bruises the next day in the third sentence. In order to understand the third sentence of this type of passage, no inference is necessary: the context of the previous two sentences directly states a cause for the bruising. An intermediately related passage, however, might only mention in the second sentence that the characters were upset. In this case, an inference is necessary in order to build a coherent representation of the meaning of the passage. Finally, in an unrelated passage, the third sentence would be completely incongruent with the previous two sentences. In response to these types of passages, controls showed context effects on the N400 to critical target words (e.g., bruises) in the final sentences, with the greatest reduction in the N400 waveform for highly related passages (the “easiest” condition, when no inference was needed), followed by intermediately related passages, and finally by the unrelated condition. In contrast, ERP results in the patient group did not distinguish among conditions in the N400 time window [44]. However, both the control and patient groups showed a similar pattern of behavioral responses to the stimuli, rating highly related passages as “very related”, followed by the intermediately related passages as slightly less related, and the unrelated passages as unrelated. In addition, although the electrophysiological response for the patient group did not differentiate among relatedness conditions in the N400 time window, patients did show a difference between related passages and unrelated passages in a later time window (700–1000 ms after reading the critical target word); in contrast, the control group did not show a difference amongst conditions in this late time window [44]. This suggests that, although patients did not show differential N400 responses depending on relatedness condition, they were attending to the task and may have attempted to integrate the targets in the unrelated condition at a later processing point compared to controls. The generation of inferences such as in the intermediately related example above is often necessary in order to properly understand discourse context, as language input often does not directly contain all of the information needed to comprehend the message. Therefore, the patient group's failure to differentiate amongst degrees of causal relatedness in the same time window as the control group suggests that schizophrenia patients have difficulty generating inferences to construct a coherent discourse representation on the same time scale as healthy adults [44].

Consistent with this pattern is another recent ERP study that presented patients and controls with five-sentence passages, each of which contained a noun that could serve as the referent of a noun in the fourth sentence but varied in terms of semantic fit [45]. For example, upon encountering *outfit* (The night before work, Lisa ironed the outfit) in the fourth sentence, possible referents mentioned in previous sentences would be *suit* (context appropriate and lexically associated), *costume* (context inappropriate but lexically associated), or *ring* (context inappropriate and lexically unassociated). The control group showed a graded ERP response throughout the N400 time window, showing the smallest amplitude for the context-appropriate/associated condition, followed by the context-inappropriate/associated condition, and finally the context-inappropriate/unassociated condition. In contrast, in the early portion of the N400 time window (300–400 ms), the schizophrenia group distinguished between globally appropriate and inappropriate semantic fit (reduced amplitude for the context-appropriate referent in comparison to either context-inappropriate referent, irrespective of lexical association). However, in the later portion of the N400 time window (400–500 ms), the pattern switched so that patients distinguished between locally associated and unassociated semantic fit (reduced amplitude for both associated conditions, regardless of context appropriateness, compared to the inappropriate-unassociated condition) [45]. In other words, the controls seemed able to benefit from the combination of context-level fit and word-level fit, whereas the schizophrenia patients seemed to be toggling between sensitivity to global and local fit [45]. 

 In summary, the literature on high-level language comprehension in schizophrenia patients shows that they are most impaired when control demands are highest, as when the use of context is needed to constrain word meaning [36] or construct a meaning at odds with the associative relations amongst individual words [37]. These results are suggestive of a role for a cognitive control deficit in abnormal language processing in schizophrenia, leading patients to fail to suppress context-irrelevant information as well as maintain linguistic context in order to guide the processing of incoming words. This pattern of results is much in line with the type of context-maintenance deficits seen in patients when performing the AX-CPT task described above, in which patients have difficulty maintaining the context of having just seen a “B” in order to respond correctly by suppressing a response to an ensuing “A” (e.g., [21]). Further, studies of discourse comprehension, a level of language processing that places relatively high demands on control mechanisms, have shown that schizophrenia patients are impaired at building coherent discourse representations [44] and are unable to effectively integrate global discourse-level context with local word-level context [45]. These studies of online discourse comprehension are supported by several offline studies showing that schizophrenia patients are unable to make use of discourse coherence when recalling discourse content [40–43]. 

The evidence reviewed above shows that deficits in discourse comprehension in schizophrenia can be accounted for by a domain-general deficit in cognitive control. As mentioned in the introduction, several theories converge on control mechanisms as related to the range of cognitive deficits seen in schizophrenia patients [5, 6, 39]. Therefore, an important question concerns specifically what aspects of control affect discourse comprehension in schizophrenia. We suggest that the current evidence is consistent with the idea that discourse comprehension deficits in schizophrenia result from (1) deficits in the controlled maintenance of context-relevant information, which may be mediated by dysfunctions of the DLPFC and (2) deficits in the ability to resolve conflicting information in the language input, and the inability to monitor the incoming input for conflict, which may be mediated by dysfunction of the ACC. Following from the Guided Activation Model discussed above, in which prefrontal regions engaged in context maintenance guide activation in the neural regions responsible for task execution, prefrontal dysfunction in schizophrenia is expected to result in processing and integration difficulties in the perisylvian-language network. Specifically, we suggest that deficits in the maintenance of context-relevant information will lead to impoverished discourse representations that are heavily reliant on word-level meaning relations. This kind of loosely integrated representation may suffice, provided incoming input does not introduce conflict. Therefore, discourse comprehension in schizophrenia patients should be most successful when the words contained in the context are related in meaning but will be more difficult for these patients when the semantic fit amongst individual words and sentences is poor.

## 5. Conclusions

In summary, we conclude that prefrontally mediated cognitive control mechanisms are impaired in schizophrenia and that during language comprehension this will most likely impact the integration and maintenance of context, which involves (especially in the case of high-level language processing) multiple levels of meaning. As context accumulates, demands on control mechanisms governing integration and maintenance are increased, resulting in discourse-level processing as a particularly demanding level of processing in terms of control. Further, as context accumulates, the potential for multiple aspects of the input to conflict increases; conflicting aspects of context maximally tap into control processes. Two main predictions are generated from this hypothesis that may be tested as part of a future research agenda using methods from the cognitive neuroscience of language: (1) schizophrenia patients will be most impaired during processing at discourse level; (2) the construction of coherent discourse representations will be most successful when supported by strong relations amongst individual words, and patients will be most impaired when aspects of the context conflict with each other.

Stemming from these predictions are several implications for future approaches to the study of high-level language comprehension in schizophrenia. First, more research on comprehension at the discourse-level is needed, with a particular emphasis on studies that deliberately manipulate sources of conflict and competition within discourse context. As noted above, sources of conflict, such as word-level ambiguity, are not special challenges to the comprehension system only found in laboratory settings, but instead are rather commonplace during natural language comprehension. Likewise, other challenges to the comprehension system, such as the generation of inferences in order to construct coherent discourse representations, are also quite common and place demands on cognitive control resources. Therefore, approaches that target those aspects of language that are most demanding of control are likely to be fruitful in the study of language deficits in schizophrenia. In addition, future emphasis on cognitive neuroscience techniques such as EEG and fMRI, particularly those that utilize connectivity analysis, will be of use in determining how maintenance and control operations in prefrontal cortex mediate language processing in schizophrenia.
